# The complete mitochondrial genome and phylogenetic analysis of the zebra turkeyfish *Dendrochirus zebra* (Cuvier, 1829) (Teleostei: Perciformes)

**DOI:** 10.1080/23802359.2026.2749909

**Published:** 2026-10-19

**Authors:** Hefang Sun, Han Lai, Wen Yu, Wenliang Zhou, Wenhua Yu, Zhiwei Zhang

**Affiliations:** a South China Biodiversity Research Center, School of Life Sciences, Guangzhou University, Guangzhou, China; b Center for Evolution and Conservation Biology, Southern Marine Science and Engineering Guangdong Laboratory (Guangzhou), Guangzhou, China; c Laoshan Laboratory Guangdong Institute, Guangzhou, China; d School of Chemistry and Materials, Guizhou Normal University, Guiyang, China

**Keywords:** *Dendrochirus zebra*, mitochondrial genome, phylogenetic analysis, Pteroinae

## Abstract

*Dendrochirus zebra*, a reef-dewelling scorpaenid, was sequenced for its complete mitochondrial genome (16,504 bp). The circular genome (52.93% A-T content) encodes 13 protein-coding genes, 22 tRNAs, two rRNAs, and one control region (D-loop). Phylogenetic analysis of 22 Scorpaenoidea species placed *D. zebra* as sister to the clade comprising *Pterois antennata* and *P. radiata*, confirming the paraphyly of the traditional genera *Dendrochirus* and *Pterois*. This molecular-morphological incongruence suggests traditional diagnostic traits are evolutionarily labile and that the venomous defensive apparatus is likely an ancestral trait rather than a convergent one. This mitogenome provides essential resources for systematic revisions of the Pteroinae.

## Introduction

Species of the genus *Dendrochirus* belong to the subfamily Pteroinae (Scorpaenidae), collectively known as lionfishes, which are top consumers in reef ecosystems controlling prey populations (Fishelson 1975). The genus is characterized by an unraised parietal spine; a ventral surface of the lower jaw devoid of ridges, spines, and scales; branched median pectoral-fin rays; and a pectoral-fin membrane extending nearly to the distal tips of the rays (Astakhov 2018). However, inter- and intrageneric relationships within the Pteroinae remain contentious due to inconsistencies between morphological traits and molecular data (Chou et al. 2023).

*Dendrochirus zebra* (Cuvier, 1829) is widely distributed throughout the Indo-Pacific region, inhabiting coral reefs, sheltered lagoons and caves (Pavlov and Emel’yanova 2019), and is listed as Least Concern (Motomura and Matsuura 2016). Venomous spines on the dorsal, anal and pelvic fins serve in defensive adaptations against predation and intrasexual competition (Moyer and Zaiser 1981). It is classified among the most dangerous venomous fish lineages bearing the SNTX toxin (Zhang et al. 2024). When threatened, it injects venom into predators, causing severe pain, swelling, and tissue necrosis (Kiriake et al. 2013). These distinctive biological traits confer substantial research potential in ecology and toxicology.

The mitochondrial genome encompasses comprehensive genetic information, confering distinct advantages in species identification and phylogenetic reconstruction (Boore 1999). To leverage these advantages, we sequenced the complete mitochondrial genome of *D. zebra* and reconstructed a phylogenetic tree to elucidate its evolutionary relationships within Pteroinae.

## Materials and methods

Specimens of *D. zebra* were collected in March 2024 from Xidao, Sanya, Hainan Province, China (18.21825° N, 109.3679165° E) (Figure 1B). Species identification was based on morphological characteristics (Figure 1A; Table S3). Muscle tissue was flash-frozen in liquid nitrogen and stored at −80 °C (accession number 2403184178; contact: luoxuanjin@gmlab.ac.cn).

**Figure 1.**
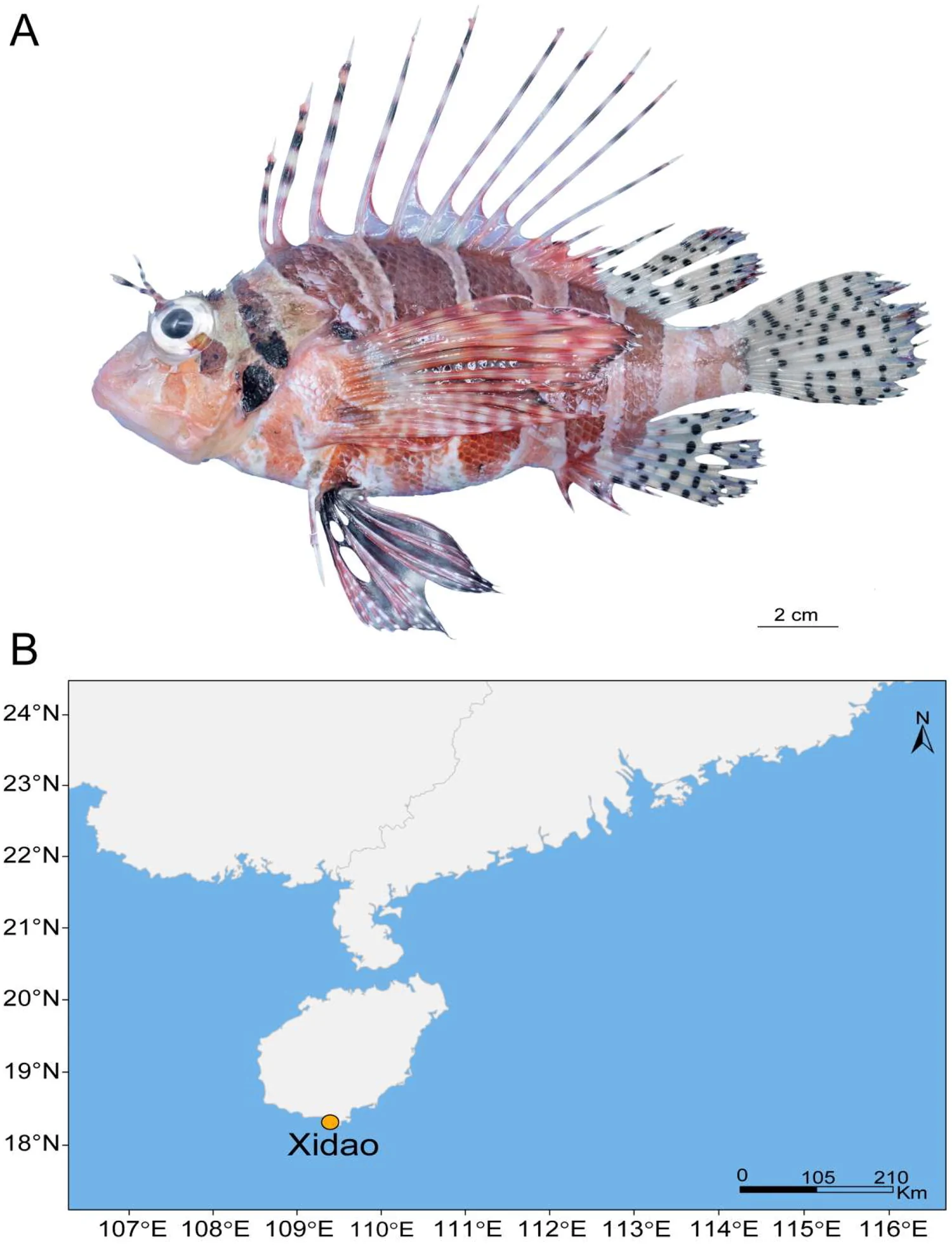
Specimen photograph and geographic distribution of the sampled individual of *Dendrochirus zebra* (QB012979). (A) Photograph of *D. zebra*, captured by Han Lai. The body exhibits a reddish coloration with 11 alternating broad and narrow dark brown transverse bands along the lateral surface; the pectoral fins extend to the posterior margin of the anal-fin base but remain proximal to the caudal peduncle; dorsal fin possesses 10–11 soft rays; the anal fin comprises of 3 spines and 6–7 soft rays; the snout bears three cirriform dermal appendages, while the supraorbital region is furnished with elongated dermal flaps; cranial spines and ridges are smooth, lacking any serrations. (B) Map showing the sampling locality (Xidao Island, South China Sea).

Genomic DNA was extracted using a modified CTAB protocol (Clarke 2009). A 300 bp insert library was constructed using the MGIEasy FS Kit and sequenced on the DNBSEQ-T7 platform (MGI-Tech, China). A total of 11.8 Gb of raw data was generated, and clean paired-end reads were obtained using Fastp v0.23.2(Chen 2023). Mitogenome assembly and annotation were performed using MitoZ v3.6 (Meng et al. 2019) with default parameters and validated using MitoFish DB Ver. 3.85 (Zhu et al. 2023). The tRNA genes were confirmed with tRNAscan-SE (Lowe and Chan 2016). Sequencing depth and coverage were assessed using BWA v0.7.17-r1188 and Samtools v1.16 (Danecek et al. 2021; Figure S1). Strand asymmetry was assessed using AT-skew and GC-skew.

Mitochondrial genomes of 21 additional Scorpaenoidea species were retrieved from the GenBank (Table S2), with *Sebastiscus marmoratus* as the outgroup (Jia et al. 2020). The 13 PCGs were extracted and sequence integrity was assessed by translation under the vertebrate mitochondrial genetic code. Individual PCGs were aligned using MUSCLE v3.8.31 (Edgar 2004) with default parameters. Ambiguous regions were filtered using trimAl v1.2 (Capella-Gutiérrez et al. 2009) (-gt 0.8 -cons 60), which removed 0.57% of sites and reduced the concatenated supermatrix from 11,493 bp to 11,427 bp.

Trimmed PCG alignments were concatenated and partitioned by gene boundaries and codon positions (1st, 2nd, and 3rd), resulting in 39 initial partitions. Maximum Likelihood (ML) phylogenetic inference was performed using IQ-TREE v1.6.12 (Nguyen et al. 2015), with optimal substitution models and partition merging strategies selected using ModelFinder (-m MFP+MERGE) based on the Bayesian Information Criterion. Topological support was assessed with 5,000 ultrafast bootstraps (UFBoot) and 1,000 SH-like approximate likelihood-ratio tests. The exact command executed was: iqtree -s concatenated_sequences.fasta -spp partitions.txt -m MFP+MERGE -bb 5000 -alrt 1000 -seed 12345 -nt AUTO -o MT789709 -pre tree. The resulting tree was visualized using iTOL (Letunic and Bork 2024).

## Results

The complete mitochondrial genome of *D. zebra* was assembled into a circular molecule at 640 × average coverage depth (Figure S1). The mitogenome showed strong sequencing support, 57.22% of nucleotide positions covered at average depth. No substantial coverage gaps were detected, indicating reliable assembly quality. The 16,504 bp mitogenome comprises 27.19% A, 25.75% T, 28.64% C, and 18.43% G, with a GC content of 38.14%. AT-skewness is 0.027 and the GC-skewness is −0.217. It encodes 13 PCGs, 22 transfer RNA (tRNA) genes, and two ribosomal RNA (rRNA) genes. Eight tRNA genes (*tRNA^Glu^*, *tRNA^Pro^*, *tRNA^Gln^*, *tRNA^Ala^*, *tRNA^Asn^*, *tRNA^Cys^*, *tRNA^Tyr^*, *tRNA^Ser1^)* and the *nd6* gene are on the light strand (L-strand); all remaining genes are on the heavy strand (H-strand) (Figure 2). All 22 tRNA genes were validated by tRNAscan-SE without discrepancy. All PCGs use canonical ATG start codon, except *cox1* (GTG). Stop codon usage varies: *nd1* and *nd6* use TAG; *cox1*, *atp8*, and *nd4l* terminate with TAA; *nd5* terminates with non-canonical AGG, while the remaining seven PCGs (*nd2*, *cox2*, *atp6*, *cox3*, *nd3*, *nd4*, and *cytb*) contain incomplete stop codons (Table S1). Three overlapping regions were identified between *atp8*–*atp6* (10 bp), *nd4l*–*nd4* (7 bp), and *nd5*–*nd6* (16 bp) (Table S1).

**Figure 2.**
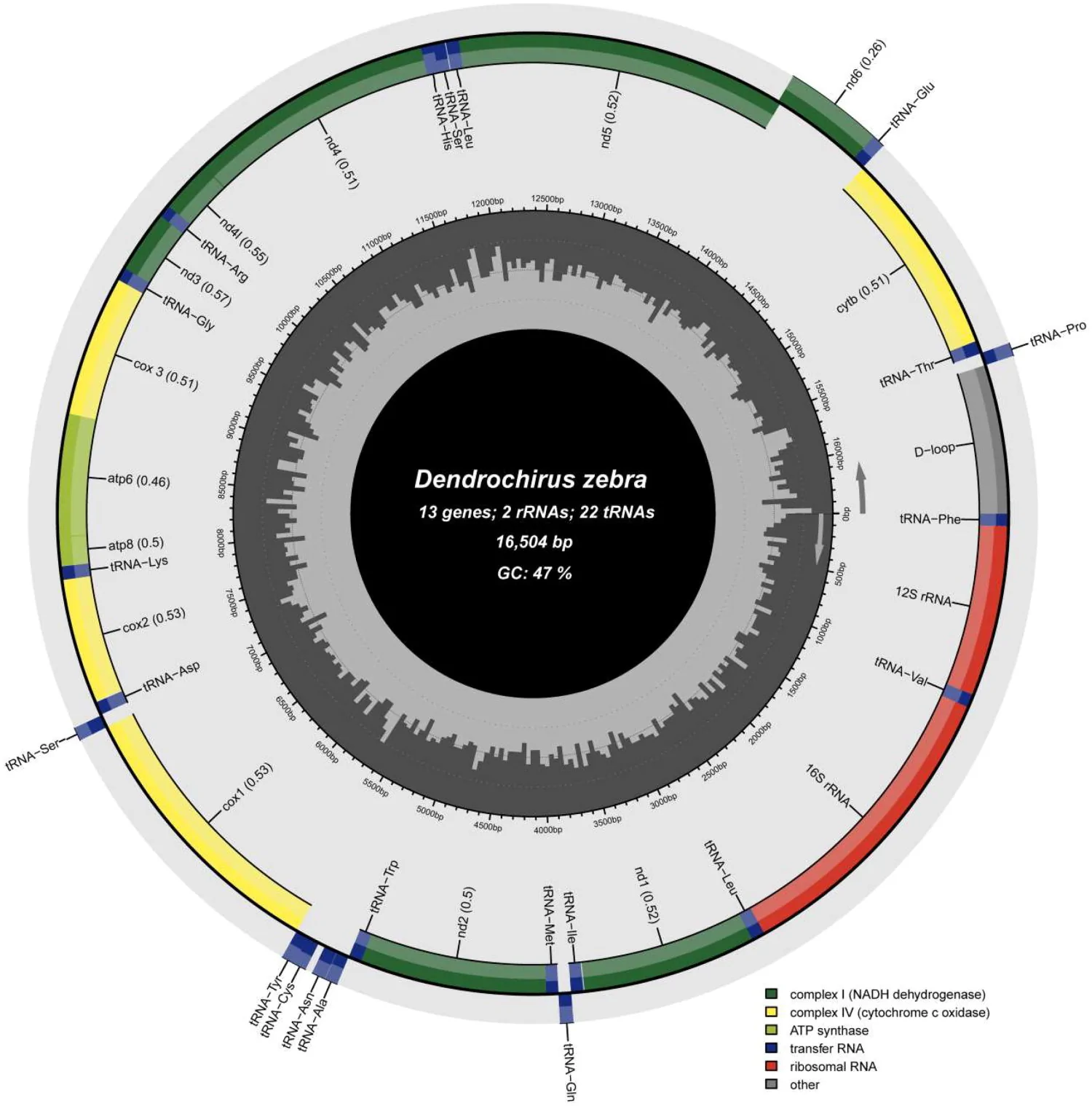
Mitochondrial genome map of *D. zebra*. Genes are marked with different colors: green for NADH dehydrogenase (*nd1-nd6* and *nd4l*), yellow for cytochrome c oxidase (*cox1-cox3* and *cytb)*, yellow-green for ATP synthase (*atp6* and *atp8)*, red for ribosomal RNA (12s rRNA and 16s rRNA) and blue for transfer RNA. The D-loop region is shown as gray. The outer ring shows the genes location, and the inner ring shows the GC content. The grey arrows indicate the orientation of gene transcription.

Phylogenetic analysis revealed that *D. zebra* did not cluster with its nominal congeners (*D. biocellatus* and *D. barberi*). Instead, *D. zebra* was sister to a clade comprising *P. antennata* and *P. radiata* (UFBoot = 100%, SH-aLRT = 100%), while *Parapterois heterurus* was sister to this three-species assemblage (Figure 3). More broadly, the phylogenetic topology explicitly revealed the paraphyly of the traditional genera *Dendrochirus* and *Pterois*. In contrast, within the subfamily Scorpaeninae, all genera were consistently recovered as well-supported monophyletic lineages.

**Figure 3.**
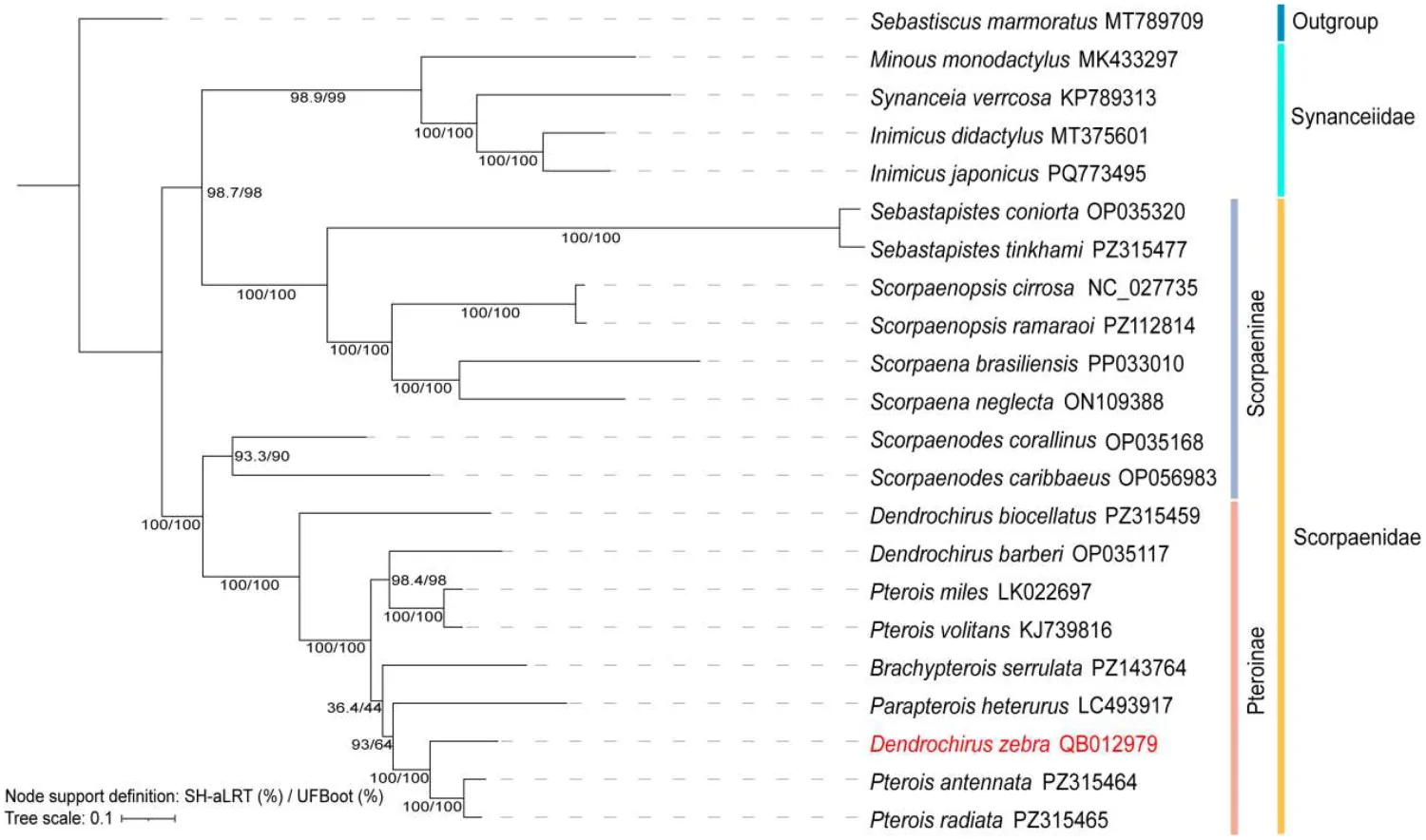
Phylogenetic trees using ML analyses based on 13 PCGs. The number on each node represent support value. Maximum likelihood (ML) phylogenetic tree of Pteroinae and related Scorpaenoidei species inferred from concatenated nucleotide sequences of 13 mitochondrial protein-coding genes (11,427 bp). The tree was constructed using IQ-TREE under an edge-unlinked partition model (-m MFP+MERGE) based on 39 initial partitions (divided by gene and codon position). Numbers at nodes represent SH-like approximate likelihood ratio test (SH-aLRT) support values (%)/Ultrafast Bootstrap (UFBoot) support values (%), respectively. *Sebastiscus marmoratus* (MT789709) was used as the outgroup. The following sequences were used: MT789709 (Jia et al. 2020), MK433297 (Kim et al. 2019), KP789313 (Wang et al. 2016), PQ773495 (Li et al. 2025), MT375601 (Kim et al. 2020), PZ315447 (unpublished), OP035320 (unpublished), PZ112814 (unpublished), NC_027735 (Wu et al. 2016), ON109388 (Patil et al. 2022), PP033010 (Alvarenga et al. 2024), OP035168 (unpublished), OP056983 (unpublished), PZ315459 (unpublished), OP035117 (unpublished), KJ739816 (Del Río-Portilla et al. 2016), LK022697 (Dray et al. 2016), PZ143764 (unpublished), LC493917 (unpublished), PZ315465 (unpublished), QB012979 (this study), PZ315464 (unpublished).

## Discussion and conclusion

The complete *D. zebra* mitochondrial genome exhibits positive AT-skew and negative GC-skew, with a conserved vertebrate mitochondrial gene organization (Alvarenga et al. 2024). Among the 13 PCGs, *cox1* utilizes the non-canonical start codon GTG, as reported in humans and certain teleosts (Saurer et al. 2023). Six PCGs demonstrate incomplete stop codons, likely completed to TAA through post-transcriptional polyadenylation (Satoh et al. 2016).

Phylogenetic analysis revealed that *D. zebra* clustered with *P. antennata*, *P. radiata*, and *P. heterurus* (Figure 3), consistent with previous reported intergeneric clustering between *Dendrochirus* and *Pterois* and questioning *Dendrochirus* monophyly (Kochzius et al. 2003). Recent taxonomic revisions have demonstrated that *Dendrochirus* and *Pterois* are polyphyletic, restricting *Dendrochirus* to its type species *D. zebra*, while other nominal species have been reassigned to distinct genera, such as *D. biocellatus* to *Nemapterois*, and *D. brachypterus* to *Neochirus* (Chou et al. 2023). Thus, our topology reflects outdated nomenclature, not a novel phylogenetic conflict. Nevertheless, as the limitation of mitochondrial genome in phylogenetic analysis, our topology must be interpreted cautiously given potential artifacts including misidentifications (Shen et al. 2013), compositional bias (Delsuc et al. 2005), alignment errors (Min and Hickey 2007), mitochondrial introgression (Toews and Brelsford 2012), and incomplete lineage sorting (Degnan and Rosenberg 2009). Independent nuclear phylogenomic evidence is needed for definitive resolution.

Traditionally, generic boundaries within Pteroinae rely on characters including pectoral-fin branching, fin-membrane, and fin spine morphology (Chou et al. 2023). The incongruence between morphological boundaries and molecular lineages suggests that these characters are evolutionarily labile, potentially reflecting convergent adaption to ecological pressures such as reef-associated ambush predation (Losos 2011). For instance, the venomous fin spines are widely distributed across *Dendrochirus*, *Pterois*, *Pteropterus*, *Nemapterois*, and *Neochirus* (Moyer and Zaiser 1981), indicating an ancestral trait with lineage-specific modifications. Whether phenotypic similarities among lineages reflect convergence or retained ancestral polymorphism requires ancestral-state reconstruction using nuclear data. In conclusion, the mitochondrial genome of *D. zebra* provides a valuable resource for phylogenetic analyses and systematic revision of the Pteroinae.

## Supplementary Material

Revised supplementary Information.docx

## Data Availability

The mitogenome is deposited in GenBank (accession QB012979). The associated BioProject, Bio-Sample and SRA number are PRJNA1471108, SAMN60440204 and SRR40630853, respectively.
