# Supplementary material for: The complete mitochondrial genome and phylogenetic analysis of the zebra turkeyfish Dendrochirus zebra (Cuvier, 1829) (Teleostei: Perciformes)
Source: Mitochondrial DNA B Resour. 2026 Oct 19;11(11):1349–53. doi: 10.1080/23802359.2026.2749909 (PMC13686219; doi:10.1080/23802359.2026.2749909)
Supplement: Supplementary file 1 [file TMDN_A_2749909_SM8803.docx]

**Supplementary Information**

**The Complete Mitochondrial Genome and Phylogenetic Analysis of the zebra turkeyfish *Dendrochirus zebra* (Cuvier, 1829) (Teleostei: Perciformes)**

Hefang Sun^1,2^, Han Lai^2,3^, Wen Yu^4^, Wenliang Zhou^2,3^, Wenhua Yu^1*^, Zhiwei Zhang^2,3*^

1 South China Biodiversity Research Center, School of Life Sciences, Guangzhou University, Guangzhou 510006, China

2 Center for Evolution and Conservation Biology, Southern Marine Science and Engineering Guangdong Laboratory (Guangzhou), Guangzhou 511458, China

3 Laoshan Laboratory Guangdong Institute, Guangzhou 511458, China

4 School of Chemistry and Materials, Guizhou Normal University, Guiyang 550001, China

* **Corresponding author**

Wenhua Yu E-mail: [wenhua_yu@gzhu.edu.cn](mailto:wenhua_yu@gzhu.edu.cn);

Zhiwei Zhang E-mail: [zhangzhiwei@gmlab.ac.cn](mailto:zhangzhiwei@gmlab.ac.cn)

Supplementary Figure S1 The coverage depth of the mitochondrial genome of *Dendrochirus zebra.*

| Gene | Position | | Size(bp) | Strand | In | Codon | | AA length |
| --- | --- | --- | --- | --- | --- | --- | --- | --- |
|  | Start | End |  |  |  | Start | Stop |  |
| *tRNA^Phe^* | 1 | 68 | 68 | H | 0 | -- | -- | -- |
| *12S rRNA* | 69 | 1016 | 948 | H | 0 | -- | -- | -- |
| *tRNA^Val^* | 1017 | 1089 | 73 | H | 0 | -- | -- | -- |
| *16S rRNA* | 1090 | 2791 | 1702 | H | 0 | -- | -- | -- |
| *tRNA^Leu^* | 2792 | 2865 | 74 | H | 0 | -- | -- | -- |
| *nd1* | 2866 | 3840 | 975 | H | -5 | ATG | TAG | 325 |
| *tRNA^Ile^* | 3846 | 3915 | 70 | H | 1 | -- | -- | -- |
| *tRNA^Gln^* | 3915 | 3985 | 71 | L | 1 | -- | -- | -- |
| *tRNA^Met^* | 3985 | 4053 | 69 | H | 0 | -- | -- | -- |
| *nd2* | 4054 | 5099 | 1046 | H | 0 | ATG | TA- | 348 |
| *tRNA^Trp^* | 5100 | 5170 | 71 | H | -1 | -- | -- | -- |
| *tRNA^Ala^* | 5172 | 5240 | 69 | L | -1 | -- | -- | -- |
| *tRNA^Asn^* | 5242 | 5314 | 73 | L | -38 | -- | -- | -- |
| *tRNA^Cys^* | 5353 | 5420 | 68 | L | 0 | -- | -- | -- |
| *tRNA^Tyr^* | 5421 | 5490 | 70 | L | -1 | -- | -- | -- |
| *cox1* | 5492 | 7042 | 1551 | H | -1 | GTG | TAA | 517 |
| *tRNA^Ser^* | 7044 | 7114 | 71 | L | -4 | -- | -- | -- |
| *tRNA^Asp^* | 7119 | 7191 | 73 | H | -5 | -- | -- | -- |
| *cox2* | 7197 | 7887 | 691 | H | 0 | ATG | T-- | 230 |
| *tRNA^Lys^* | 7888 | 7962 | 75 | H | -1 | -- | -- | -- |
| *atp8* | 7964 | 8131 | 168 | H | 10 | ATG | TAA | 56 |
| *atp6* | 8122 | 8804 | 683 | H | 0 | ATG | TA- | 227 |
| *cox3* | 8805 | 9589 | 785 | H | 0 | ATG | TA- | 261 |
| *tRNA^Gly^* | 9590 | 9661 | 72 | H | 0 | -- | -- | -- |
| *nd3* | 9662 | 10010 | 349 | H | 0 | ATG | T-- | 116 |
| *tRNA^Arg^* | 10011 | 10079 | 69 | H | 0 | -- | -- | -- |
| *nd4l* | 10080 | 10376 | 297 | H | 7 | ATG | TAA | 99 |
| *nd4* | 10370 | 11750 | 1381 | H | 0 | ATG | T-- | 460 |
| *tRNA^His^* | 11751 | 11819 | 69 | H | 0 | -- | -- | -- |
| *tRNA^Ser^* | 11820 | 11887 | 68 | H | -4 | -- | -- | -- |
| *tRNA^Leu^* | 11892 | 11964 | 73 | H | 0 | -- | -- | -- |
| *nd5* | 11965 | 13815 | 1851 | H | 16 | ATG | AGG | 617 |
| *nd6* | 13800 | 14321 | 522 | L | 0 | ATG | TAG | 174 |
| *tRNA^Glu^* | 14322 | 14390 | 69 | L | -6 | -- | -- | -- |
| *cytb* | 14397 | 15537 | 1141 | H | 0 | ATG | T-- | 380 |
| *tRNA^Thr^* | 15538 | 15609 | 72 | H | 1 | -- | -- | -- |
| *tRNA^Pro^* | 15609 | 15678 | 70 | L | 0 | -- | -- | -- |
| *D-loop* | 15679 | 16504 | 826 | H | 0 | -- | -- | -- |

Supplementary Table S1 Genomic characteristics of the complete mitochondrial genome of *Dendrochirus zebra* (QB012979). In intergenic nucleotides, positive values indicate overlap and negative values denote gaps; H and L indicate heavy and light strands.

| Species | Family | Subfamily | GenBank | Accession | Length | Publication status | Reference |
| --- | --- | --- | --- | --- | --- | --- | --- |
| *Sebastiscus marmoratus* | Sebastidae | Sebastinae | MT789709.1 | MT789709 | 17,580 bp | Biosci Rep (2020) In press | Jia et al., 2020 |
| *Minous monodactylus* | Synanceiidae | Minoinae | MK433297.1 | MK433297 | 16,459 bp | Mitochondrial DNA B Resour 4 (1), 1377-1378 (2019) | Kim et al., 2019 |
| *Synanceia verrucosa* | Synanceiidae | Synanceiinae | KP789313.1 | KP789313 | 16,506 bp | Mitochondrial DNA, 1-2 (2015) In press | Wang et al., 2016 |
| *Inimicus didactylus* | Synanceiidae | Choridactylinae | PQ773495.1 | PQ773495 | 16,670 bp | Mitochondrial DNA B Resour 10 (4), 304-308 (2025) | Li et al, 2025 |
| *Inimicus japonicus* | Synanceiidae | Choridactylinae | MT375601.1 | MT375601 | 16,978 bp | Mitochondrial DNA B Resour 5 (3), 3159-3160 (2020) | Kim et al, 2020 |
| *Sebastapistes coniorta* | Scorpaenidae | Scorpaeninae | OP035320.2 | OP035320 | 16,874 bp | Unpublished | - |
| *Sebastapistes tinkhami* | Scorpaenidae | Scorpaeninae | PZ315477.1 | PZ315477 | 16,620 bp | Unpublished | - |
| *Scorpaenopsis ramaraoi* | Scorpaenidae | Scorpaeninae | PZ112814.1 | PZ112814 | 16,970 bp | Unpublished | - |
| *Scorpaenopsis cirrosa* | Scorpaenidae | Scorpaeninae | - | NC_027735 | 16,966 bp | Mitochondrial DNA, 1-2 (2015) In press | Wu et al, 2016 |
| *Scorpaena neglecta* | Scorpaenidae | Scorpaeninae | ON109388.1 | ON109388 | 17,202 bp | Mitochondrial DNA B Resour 7 (1), 1375-1376 (2022) | Patil et al., 2022b |
| *Scorpaena brasiliensis* | Scorpaenidae | Scorpaeninae | PP033010.1 | PP033010 | 16,756 bp | BMC Genomics 25 (1), 215 (2024) | Alvarenga et al, 2024 |
| *Scorpaenodes caribbaeus* | Scorpaenidae | Scorpaeninae | OP056983.1 | OP056983 | 16,496 bp | Unpublished | - |
| *Scorpaenodes corallinus* | Scorpaenidae | Scorpaeninae | OP035168.1 | OP035168 | 16,523 bp | Unpublished | - |
| *Dendrochirus biocellatus* | Scorpaenidae | Pteroinae | PZ315459.1 | PZ315459 | 16,501 bp | Unpublished | - |
| *Brachypterois serrulata* | Scorpaenidae | Pteroinae | PZ143764.1 | PZ143764 | 16,492 bp | Unpublished | - |
| *Dendrochirus barberi* | Scorpaenidae | Pteroinae | OP035117.1 | OP035117 | 16,501 bp | Unpublished | - |
| *Pterois miles* | Scorpaenidae | Pteroinae | LK022697.1 | LK022697 | 16,497 bp | Mitochondrial DNA A DNA Mapp Seq Anal 27 (1), 783-784 (2016) | Dray et al, 2016 |
| *Pterois volitans* | Scorpaenidae | Pteroinae | KJ739816.1 | KJ739816 | 16,500 bp | Mitochondrial DNA, 1-2 (2014) In press | Del Río-Portilla et al, 2016 |
| *Parapterois heterurus* | Scorpaenidae | Pteroinae | LC493917.1 | LC493917 | 16,475 bp | Unpublished | - |
| *Pterois radiata* | Scorpaenidae | Pteroinae | PZ315465.1 | PZ315465 | 16,496 bp | Unpublished | - |
| *Dendrochirus zebra* | Scorpaenidae | Pteroinae | QB012979.1 | QB012979 | 16,504 bp | This study | This study |
| *Pterois antennata* | Scorpaenidae | Pteroinae | PZ315464.1 | PZ315464 | 16,496 bp | Unpublished | - |

Supplementary Table S2 species from NCBI used for phylogenetic tree creation.

| Morphological characters | This study (Voucher No. 2403184178) | Fishbase | Marine fishes of China | The fish database of Taiwan |
| --- | --- | --- | --- | --- |
| Dorsal spines and dorsal soft rays | 13; 10 | 13; 10-11 | 13; 10-11 | 13; 10-11 |
| Anal spines and anal soft rays | 3; 6 | 3; 6-7 | 3; 6 | 3; 6-7 |
| Pectoral fins and morphology | 17; Pectoral fin Broad and long, extending backward past anal-fin origin; upper rays branched | 17; - | -; - | -; Pectoral fins broad and long, extending to posterior end of anal-fin base, with upper rays branched. |
| Pelvic-fins | 1; 5 | - | 1; 5 | - |
| Body color and transverse bands | Body reddish, with 11 alternating broad and narrow brownish transverse bars on side. | Body reddish with 5 dark bars, alternating with thin dark bars in large specimens | Body reddish, with 11 alternating broad and narrow brownish transverse bars on side. | Body reddish with 5 dark bars, alternating with thin dark bars in large specimens |
| Rostral barbel | Tip of snout with 3 flap-like barbels. | - | Tip of snout with 3 flap-like barbels. | Snout with 3 short barbels. |
| Other | Lower part of operculum with a large black spot.; soft dorsal, caudal, and anal fins covered with dark spots | Dark spot on cheek; median fins with small dark spots | Lower part of operculum with a large black spot.; dorsal, caudal, anal, and pectoral fins with multiple rows of articulated spots. | Dark blotches on check.; dorsal fin spines with longitudinal series of dark brown spots; soft dorsal, anal, and caudal fins all pale, scattered with dark spots. |

Supplementary Table S3. Diagnostic morphological and meristic characters of the sequenced voucher specimen (No. 2403184178) compared with three designated taxonomic descriptions for *Dendrochirus zebra*.
